# Clinical experience of omani undergraduate nursing students: Qualitative study

**DOI:** 10.1016/j.heliyon.2023.e20332

**Published:** 2023-09-22

**Authors:** Zeinab AlAzri, Asma Al Yahyaei, Arwa Atef Obeidat, Jahara Hayudini

**Affiliations:** a Maternity and Child Health Department, College of Nursing, Sultan Qaboos University, Muscat, Sultanate of Oman; b Fundamental and Administration Department, College of Nursing, Sultan Qaboos University, Muscat, Sultanate of Oman; c Freelancer Nurse Consultant, Europe, Sultanate of Oman

**Keywords:** Nursing, Clinical practice, Clinical education, Qualitative study

## Abstract

**Background:**

Commencing clinical practice is a main milestone in the educational journey of undergraduate nursing students as it indicates the beginning of their professional life. The clinical training experience is frequently described as stressful and challenging for nursing students.

**Objective:**

s: This study was conducted mainly to examine, explore and interpret nursing students' perceptions of the challenges they faced as nursing students during their clinical experience.

**Setting:**

College of Nursing at Sultan Qaboos University, Oman.

**Participants:**

A total of 32 undergraduates nursing students who were enrolled in the nursing program and had finished at least one clinical course from their study plan.

**Method:**

Data were collected using focus group discussions. A total of 32 participants were recruited and six focus groups were conducted. Data was transcribed and it was analyzed using thematic analysis.

**Result:**

Two main themes were identified, and under each theme, several subthemes were merged. The two main themes are: challenges that hindered self-directed learning, which included instructor approach and nurse approach, and challenges that hindered experiential learning, which included theory-practice gap, insufficient practice, lack of confidence and evaluation methods.

**Conclusi:**

on: This study provided insights into the challenges that hindered the effectiveness of clinical nursing education. Several recommendations were proposed to maximize the benefits of clinical practice and to create less stressful environments.

Globally, nursing is learned by doing, it is a practice-based discipline as noted by Ref. [1]. Clinical education is an integral component of all undergraduate nursing programs [2]. The primary advantages of the clinical education are that it allows students to translate theory into practice and prepares student nurses to understand and apply clinical principles. Clinical education also encourages students to employ critical thinking skills [3]. However, learning in a clinical setting does not come without some challenges, for example, those associated with a lack of control over the clinical area [1,2]. These challenges limit the student's ability to learn and increases the burden on the clinical instructor to secure an effective learning environment. In the literature, the main identified challenges by nursing students during their clinical practice were anxiety, theory-practice gap and clinical instructors [[1], [2], [3], [4]].

There is an agreement in the literature that initial clinical experience was the most anxiety-generating element of nursing students' clinical experience [1,3,5,6]. The main sources of this anxiety expressed by nursing students include the complexity of the clinical environment, unfamiliar areas, demanding patients, fear of making mistakes and being evaluated by teachers. Students identified role confusion as a major cause of their stress because students do not know what their role is in such an environment and become confused trying to meet the teachers, nurses and patients’ expectations [1,6]. According to the study by Papthanasiou and colleagues (2014), there is a noticeable gap between the expectations and reality of the clinical learning environment in a clinical setting which could lead to more stress and confusion.

Another identified global concern related to clinical education was the theory-practice gap. According to Ref. [1] this gap was not created because of a lack of theoretical knowledge, but due to the lack of students' abilities to apply them in a clinical setting with real patients. Researchers have seen several instances when students, although having the necessary theoretical understanding, struggle at the patient's bedside and are unable to independently perform the tasks [4]. Unfortunately, the negative impact of the theory-practice gap in nursing education was found to be persistent during the transition period of newly graduated nurses from Oman. It contributed to an increased reality shock in which new graduates found themselves unable to utilize their theoretical knowledge in practice [7].

Clinical instructors play a vital role in effective clinical education if they pose certain characteristics like good interpersonal relationships with students, approachability, competency, and good teaching skills [8,9]. Unfortunately, without these characteristics, the clinical instructor has been identified as a potential threat to students’ clinical experience. Students perceived incompetent clinical instructors as a major challenge that hindered clinical practice. Students reported that inadequate mastery of clinical skills, lack of feedback from their clinical instructors, and low teaching skills diminished the effectiveness of their clinical exposure. In addition, students reported that the great emphasis on evaluation in clinical training by the teachers provided a sense that it is the most important part of the educational process ([10].

There is a substantial body of literature regarding clinical learning environments, but very few studies have explored the perception of nursing students of their clinical experiences, particularly in Oman. Currently, nursing is a young emerging profession in the Sultanate of Oman with more than 50% of the workforce being expatriates according to published statistics from the [11]. The country has invested in nursing education to prepare local nursing graduates to take the lead in developing the profession.

As the profession of nursing continues to evolve, the educational experiences and opportunities for learning in the clinical setting will need further restructuring to complement the attributes of nursing students who wish to be a nurse. Thus, to enhance and improve the quality of clinical education, it is necessary to constantly evaluate the current situations, to identify the strengths and the areas for improvement by examining the perception of the nursing students. Despite the fact that some studies have been done worldwide to explore nursing students' perceptions of their clinical experience, the transferability of their findings to the clinical setting in Oman is limited. This limitation is due to the effects of the clinical settings and instructors' culture and approach, which vary between different countries in nursing education. For example, many studies found that clinical placement is a big challenge for the nursing students. Such challenge might not be applicable to students as there are many hospitals available for clinical placement [4,8,9,12]. It is important to note that Oman is investing in nursing education and striving to meet international standards. For instance, the minimum requirement for instructors teaching in bachelor programs is a master's degree. These efforts highlight the commitment of Oman to enhance the quality of nursing education and align it with global benchmarks. This study aims to explore the clinical experience of Omani undergraduate nursing students using focus groups. The knowledge to be gained from this study will expand our understanding of the factors that affect nursing students' clinical experience. Hence, such understanding will be a scientific base for future efforts to enhance the quality of clinical education.

## 1 Method

### 1.1 Design

This article reports findings from the focus groups’ narratives. Qualitative description design guided the data collection and analysis of this study.

### 1.2 Setting

This study was conducted at the College of Nursing at SQU in Muscat in Oman. The College of Nursing was opened in 2009. Since then the college has established 4 nursing program(1 Bachelor and 3 Master degree). It enrolls approximately 120 undergraduate nursing students each year in its five-year program from all over the country. Around 80% of the enrolled students are females. In this program, English is the language of instruction. English language preparation and theoretical courses are taught in the first two years and clinical courses start in the third year. The theories of adult learning are integrated into each course of the program. These theories provide key principles on how adults learn, including self-direction, transformation, experience, mentorship, mental orientation, motivation and readiness to learn [13].

### 1.3 Participants

A convenience sampling technique was utilized as it is cost-effective, readily available and suitable for exploratory design [14]. The eligibility criteria were (a) nursing students, (b) registered for the BSN program at the College of Nursing at SQU and (c) enrolled in at least two clinical courses. The exclusion criteria were (a) bridging students who already have a diploma in nursing, (b) students who have less than two clinical courses and (C) students were not registered in courses with any member of the research team. According to Ref. [15]; five or six focus groups are adequate to attain information redundancy.

### 1.4 Data collection procedure

The study obtained ethical approval by the College of Nursing, SQU. The research team members worked together in data collection and analysis. The first and second authors are Omani nurse educators, therefore, they have insight into the Omani cultural nuances. The rest of the research team has significant experience in nursing education in Oman.

The principal investigator (PI), the first author of this article, sent a study invitation through the college administration to all the nursing students who had completed two clinical courses. The invitation included information regarding the study. Although the potential participants were directed to a link to register, they also contacted the PI directly to register. The PI ensured that recruited students were not registered in courses with any member of the research team. All potential participants registered in groups with their classmates. The researchers screened the participants according to the eligibility criteria before they confirmed the group members’ participation. Students in each group decided on a convenient time for the group discussion that did not conflict with their classes. All focus groups were conducted at the college, in a private psychiatric lab with the door closed.

At the beginning of each focus group, each participant signed an informed consent and completed a demographic survey. The survey included questions about age, academic year, gender and the number of completed clinical courses. A semi-structured interview guide was used by the moderator to guide the discussion. The interview guide questions are listed in Table 1.

**Table 1:** Interview guide questions.

1	Tell me how you feel about being a nursing student.
2	Tell me how you feel about your clinical experience.
3	Tell me what you think about your clinical experience.
4	Tell me about your suggestions to improve your clinical experience

Additionally, the focus group discussions were conducted in a controlled and confidential setting within the college premises. The private psychiatric lab was chosen specifically for its suitability in maintaining the privacy of participants and creating a conducive environment for open and honest responses. The door to the lab was closed during the discussions to minimize external distractions and maintain the confidentiality of the conversations. By providing a controlled and confidential environment, we aimed to create a safe space for participants to freely express their perceptions and experiences related to the clinical setting. This approach allowed for a more accurate and honest representation of the participants' viewpoints, contributing to the validity and reliability of the study findings.

The PI moderated all six focus group discussions. She introduced herself and her credentials at the beginning of each group discussion. One of the research team members took notes regarding the body language and group dynamics that could affect the shared experience. All discussions were audio-recorded. The group discussions were conducted in English. At the end of each discussion, the PI summarized the main findings from the discussion in the form of member checking to confirm the accuracy of her understanding.

### 1.5 Ethical considerations

Approval for the study was obtained from the Ethics Research Committee of the College of Nursing,at Sultan Qaboos University(SQU). Prior to the interviews, participants provided written consent, and strict confidentiality was upheld throughout the process. Audio recordings were securely stored in a locked location under the supervision of the principal/co-principal investigators at the college. Research data was kept in a secure cabinet within the College of Nursing, with exclusive access granted to the principal/co-principal investigators. The data will be retained for a period of five years, after which it will be disposed of in accordance with institutional regulations.

### 1.6 Trustworthiness

Several techniques were utilized throughout this study to augment the (rigor and trustworthiness. First, the triangulation of different focus groups and literature reviews were done. The second technique used was member checking at the end of each discussion to ensure the accuracy of the moderator's understanding and students were asked to validate the summary. Third, the PI wrote a weekly reflection on her assumptions, experience, expectations and feelings. An audit trail was maintained by the PI for the study details and progress. This report aligned with the Standard for Reporting Qualitative Research protocol (SRQR) [16].

### 1.7 Data analysis

A thematic analysis was conducted. Each group was treated as a unit of analysis. Each researcher conducted a manual line-by-line analysis independently, then codes were compared during (frequent meetings and incongruences resolved with discussions. Initially, within-group analysis was conducted, then cross group analysis was also performed. The code list was finalized based on both analyses. After that, codes were merged into overarching themes. Themes were labeled to reflect the accurate meanings.

## 2 Results

The sample characteristics are presented in Table 2. Six focus groups were conducted with a total of 32 participants, five to six participants per group. The focus group's duration ranged between 60 and 90 min. The participants' ages ranged from 20 to 23 years old. All participants were in their third to fifth year of the nursing program. Four focus groups included only female participants and two groups included only male participants. This ratio is consistent with the female to male nursing student ratio at the college.

**Table 2:** Descriptive information of participants (32).

Group	N	Gender	Cohort	Age (years)
1	6	Males	Fifth year	23–24
2	5	Females	Fourth year	22
3	5	Females	Fifth year	23
4	5	Females	Fifth year	23
5	5	Females	Fourth year	22
6	6	Males	Fourth year	22–23

There are two overarching themes with multiple sub-concepts within each theme. The themes and subthemes are illustrated in Table 3.

**Table 3:** Themes and sub-concepts.

Theme	Sub-concepts
**Challenges hindered Self-directed learning**	● Instructor approach ● Nurse approach
**Challenges hindered Experiential Learning**	● Theory practice gap ● Insufficient practice ● lack of confidence ● Evaluation methods

## 3 Theme 1: Challenges that hindered self-directed learning

In the self-directed learning theme, the participants described their perspectives on being instructor-led versus being more self-directed in the clinical learning process. The two sub-concepts within this theme were the instructor approach and the nurse approach.

### 3.1 instructor approach

The students in all six groups described how instructors created the learning environment. The learning environment could be stressful and controlling or a comfortable and trusting environment. One participant said, “I think the approach of the clinical instructor is sometimes what makes me stressed.” Another participant said, “The instructor allowed us to give IM vaccine to children which makes me feel so happy. On the other hand, some instructors never trust you.”

Student participants also perceived that some instructors limited the amount of student freedom to take certain decisions in the clinical setting. One student said, “Once you entered the clinical setting, you feel your freedom is restricted.” All six groups discussed how their freedom was restricted by providing many examples. These examples included the type of procedures the students needed to perform, time spent with patients and the need for the instructor's presence to perform procedures. One student provided an example by saying, “Even if we go out of the patient's room, the instructor insists that we go to stay with the patient. One time I told the instructor that the patient is sleeping, and she insists that I have to stay with the patient.”

Furthermore, participants discussed how the teaching methods used by the instructors limited their freedom during the clinical day. For example, presenting a case study with PowerPoint slides that required a lot of data collection from the hospital system, assigning each student to only one patient without any group discussion, and filling out many forms throughout the clinical day. This restriction was emphasized by a participant who said, “In the clinical day, the focus is on data collection from the computer for the presentation, then we go to the patients to collect more data. By this, the student does not gain practical skills which affects our clinical experience.”

### 3.2 Nurse approach

Most groups explained that most nurses trusted the students and provided them with a safe environment to perform many procedures. However, two groups discussed the feeling that nurses from some hospitals undervalued SQU nursing students. These students felt that nurses believed that nursing students from other institutions were more skillful. For example, one participant stated, “I feel so upset to mention that I am a nursing student at SQU. Some nurses said that we [SQU students] only have knowledge, no skills.” This indicated the mistrust some students experienced and how this mistrust hindered their self-directed learning experience.

## 4 Theme 2: Experiential learning

In this theme, participants described how their clinical education did not provide them with sufficient practical experience. The four sub-concepts entailed within this theme were the theory-practice gap, insufficient practice, lack of confidence and evaluation methods.

### 4.1 Theory-practice gap

All participants reported that they have sufficient knowledge about the theoretical aspects of diseases and patient care. One student made this statement, “Regarding knowledge, we think that we are qualified and have sufficient knowledge.” However, the participants claimed that although they have sufficient knowledge, there was a gap between theory courses and clinical practice. The statement, “We have the knowledge, but when we try to apply it, we don't know what to apply.” This statement indicated the perceived gap between theory and practice. Also, they mentioned that sometimes they went to clinical areas where they encountered diseases that they had not learned about in the theory course.

### 4.2 Insufficient practice

All six groups discussed the lack of sufficient practice during clinical days. They defined the practice as performing many procedures multiple times to master them. One student said, “I have no chance to practice most of the procedures such as intra-muscular injection, and some procedures we did only a few times.” The participants identified multiple reasons for this insufficient practice. The length of exposure in clinical areas was the most reported reason for the insufficient practice. As evidence, one student said, “The time allocated for the clinical [practice] is too short.” All groups agreed that spending one day per course per week (8 h/week/course) from 8 a.m. to 4 p.m. was not enough for them to practice procedures, especially because most patients take naps between 1 and 4 p.m. In addition, the students become exhausted and cannot focus during such a long clinical day. They preferred the two-day system, in which there were two days from 8 a.m. to 12 p.m. per course per week. In such a system, there are more chances for procedures to be performed, and the students were more focused and active. Furthermore, the student to instructor ratio, which was 6:8, student per instructor, was perceived by the participants in three groups as a reason for insufficient practice. Also, the focus of the instructors is more on theoretical aspects of patient care as illustrated by the participant's statement, “The main focus of the clinical instructor is on the theory, but at the time we need more focus on the skills.” This is another cause of insufficient practice. Limiting each student to care for only one patient was also one of the reasons mentioned for the perceived insufficient practice mentioned by one student who said, “The main reason is that they assign us to only one patient. If it was two patients or more, then we will perform more procedures and we will benefit more.”

### 4.3 Lack of confidence

Most of the participants expressed their feelings of a lack of confidence. This lack was linked directly to insufficient practice. One participant noted that, “We have the knowledge, but we lack confidence, we need to be more confident.” Another participant also mentioned that, “At the beginning of this semester I was thinking about how I will do any procedures or give IM injection when I graduate, honestly, I have no confidence.”

### 4.4 Evaluation methods

The students perceived the purpose of clinical education as evaluative more than educational. Participants perceived that evaluation was more important than teaching for the instructors, as illustrated by one participant who said, “Our instructors focus on identifying our mistakes more than on teaching us.” The participants emphasized that too many assignments, most of them paperwork, in clinical courses limited their experiential learning. One participant illustrated this thinking by saying, “The instructors evaluate us based on our paperwork, not clinical performance, to be honest, we have ready-made care plans, and we keep re-utilizing them every semester.”

## 5 Discussion

Our findings describe the clinical experience of Omani undergraduate nursing students at SQU. To the best of our knowledge, this study is the first published study exploring the clinical experience of Omani nursing students.

The integration of adult learning theories in designing any adult education program is essential to enhance learning effectiveness. Although nursing clinical education at SQU is built based on the principles of adult learning theories, the findings of this study indicate that the desired result of such integration is not yet achieved. Students in the current study perceived many challenges that impede their clinical learning to be self-directed or experiential. This finding is consistent with the findings of other studies exploring nursing students’ experience in clinical education [3,[17], [18], [19]].

It could be speculated that the reason students did not experience their clinical learning as self-directed or experiential, despite the integration of these principles in the nursing program, is due to improper implementation of these principles in the nursing education context. The instructor approach and nurse approach sub-concepts within self-directed learning could explain how instructors and nurses create a controlling environment that does not support self-directed learning [3,[17], [18], [19]]. Similar findings was found by Ref. [12] in their recent study which revealed that students felt empowered when instructors encouraged self-direction and students appreciated positive role modeling by their instructors. Improper implementation, as evidenced by insufficient practice and the theory-practice gap, could also lead to the ineffectiveness of experiential learning. Although nursing clinical hours and the student to instructor ratio at SQU are based on international standards to ensure sufficient practice, the proper utilization of clinical time should be investigated further. Therefore, a future study with a community participatory design that involves nurses, nursing instructors and students could provide an in-depth understanding of the challenges that could hinder students’ learning. Such a study could guide nursing instructors to reevaluate their teaching techniques and evaluation methods. In addition, an interventional study with training programs for instructors and nurses about the effective implementation of theories of adult learning would be helpful to improve skills and approaches with the students in clinical settings.

Unrealistic expectations of nursing students from their clinical education could be another justification for their description of their learning as not self-directed nor experiential. Although the clinical education at SQU is internationally accredited and the nursing program's clinical hours are internationally accepted, the students still perceived the practice as insufficient. Their perceptions of insufficient practice led them to feel incompetent and unconfident. The findings of this study are in line with the findings of other studies [20]. The mismatch between nursing students' expectations and the reality has led to nursing students dropping out of schools [21]. As mentioned before, a study with a community participatory design that involves students would further enrich our understanding of how these unrealistic expectations could hinder students' learning effectiveness. In addition, an interventional study that implements an orientation program about the real expectations for nursing students at each level is advocated.

## 6 Limitations

Limitations of this study need to be considered. In terms of generalizability, the findings of this study may not reflect the experience of nursing students in other nursing colleges in Oman. However, the researchers collected rich descriptions which make the current study transferable to other Omani contexts. Furthermore, the focus group discussions were moderated by instructors who were known to the students, so there was a chance the students responded in a way to be seen in a positive light. Although confidentiality and privacy were provided to the students during the discussion and they were assured that their participation would not affect their grades, the chance of responding in a way to gain social desirability can not be excluded. Moreover, this study is limited to exploring students' perspectives about their clinical experience, therefore, to obtain a holistic understanding of the challenges, nursing instructors' and nurses’ perspectives should be explored in future studies.

## 7 Implementations

A future interventional study with a community participatory research approach is suggested. Such research will provide us with an in-depth understanding of the factors that hinder the effectiveness of clinical education from students, instructors and nurses’ perspectives. Hence, this understanding will be valuable in designing training programs for nursing students based on their true needs. Moreover, the obtained knowledge would help stakeholders reevaluate nursing clinical education to enhance its effectiveness.

## 8 Conclusion

This study provides insights into the challenges that hinder the effectiveness of clinical nursing education. These challenges were classified into two themes: challenges that hinder self-directed learning and challenges that hinder experiential learning. Based on the findings of this study, an interventional study with a training program for students and instructors is advocated. Also, a study utilizing a community participatory design to gain input about clinical education challenges from students, nursing instructors and nurses’ perspectives is recommended.

## Author contribution statement

Zeinab Al-Azri; Asma Al Yahyaei: Conceived and designed the experiments; Performed the experiments; Analyzed and interpreted the data; Contributed reagents, materials, analysis tools or data; Wrote the paper. Arwa Obeidat: Performed the experiments; Analyzed and interpreted the data.Jahara Hayudini: Conceived and designed the experiments; Performed the experiments; Analyzed and interpreted the data.

## Data availability statement

Data will be made available on request.

## Declaration of competing interest

The authors declare the following financial interests/personal relationships which may be considered as potential competing interests:Dr Asma Al Yahyaei reports a relationship with Sultan Qaboos University that includes: employment.
